# Plasma acylcarnitine levels increase with healthy aging

**DOI:** 10.18632/aging.103462

**Published:** 2020-06-16

**Authors:** Zachery R. Jarrell, M. Ryan Smith, Xin Hu, Michael Orr, Ken H. Liu, Arshed A. Quyyumi, Dean P. Jones, Young-Mi Go

**Affiliations:** 1 Division of Pulmonary, Allergy and Critical Care Medicine, Atlanta, GA 30322, USA; 2 Division of Cardiology, Emory University School of Medicine, Atlanta, GA 30322, USA

**Keywords:** aging, carnitine, lipid metabolism, mitochondria

## Abstract

Acylcarnitines transport fatty acids into mitochondria and are essential for β-oxidation and energy metabolism. Decreased mitochondrial activity results in increased plasma acylcarnitines, and increased acylcarnitines activate proinflammatory signaling and associate with age-related disease. Changes in acylcarnitines associated with healthy aging, however, are not well characterized. In the present study, we examined the associations of plasma acylcarnitines with age (range: 20-90) in 163 healthy, non-diseased individuals from the predictive medicine research cohort (NCT00336570) and tested for gender-specific differences. The results show that long-chain and very long-chain acylcarnitines increased with age, while many odd-chain acylcarnitines decreased with age. Gender-specific differences were observed for several acylcarnitines, e.g., eicosadienoylcarnitine varied with age in males, and hydroxystearoylcarnitine varied in females. Metabolome-wide association study (MWAS) of age-associated acylcarnitines with all untargeted metabolic features showed little overlap between genders. These results show that plasma concentrations of acylcarnitines vary with age and gender in individuals selected for criteria of health. Whether these variations reflect mitochondrial dysfunction with aging, mitochondrial reprogramming in response to chronic environmental exposures, early pre-disease change, or an adaptive response to healthy aging, is unclear. The results highlight a potential utility for untargeted metabolomics research to elucidate gender-specific mechanisms of aging and age-related disease.

## INTRODUCTION

Age is the number one risk factor for several human health issues, those referred to as age-related diseases [1, 2]. These age-related diseases grow in importance as the median age of the world’s population continues to increase [3, 4]. As a result, the molecular mechanisms influencing aging have long been a topic of interest for study [5–7]. Recent developments in the study of the human metabolome have allowed for wide-spread study of changes in the metabolome in association with aging and age-related disease [1, 8, 9].

Acylcarnitines are carrier forms of fatty acids required for import of long-chain (LC) fatty acids into mitochondria for β-oxidation to occur [10]. The most common reported changes is an increase in blood concentration of LC acylcarnitines in individuals with age-related diseases [11–14]. Dysregulation of acylcarnitine homeostasis has been tied to a variety of age-related diseases, including cardiovascular disease [11, 12, 15], type II diabetes mellitus [13, 16, 17], osteoarthritis [18], chronic obstructive pulmonary disease [19], macular degeneration [14], glaucoma [20] and Alzheimer’s disease [21–23]. In addition to the association with age-related diseases, abnormal acylcarnitine levels are associated with activation of inflammation [24] and mitochondrial dysfunction [25, 26]. Loss of mitochondrial function both contributes to the process of aging and is, itself, an indirect result of aging. Mitochondrial dysfunction has been documented to play a role in development of most age-related diseases; however, it occurs independently of disease [27–29]. Additionally, healthy older individuals require more time to reestablish acylcarnitine homeostasis after stimulation with insulin, and higher acylcarnitine levels are found in aged men with reduced physical ability when compared with similarly aged men with normal physical ability [30, 31]. Gender differences in acylcarnitine associations with age have not been well studied, but some differences between men and women have been reported [32, 33]. Additionally, gender differences in lipid metabolites related to lifespan have been reported [34].

Neither LC acyl-CoA nor free LC fatty acids can migrate across the inner membrane of the mitochondria, so the formation of acylcarnitines is critical to metabolism of LC fatty acids [35]. This system, termed the carnitine shuttle [see Reuter & Evans [25] for review], normally maintains carnitine and acylcarnitine within a narrow range [35]. This ensures normal functioning of fatty acid β-oxidation as well as adequate availability of CoA. Acylcarnitines are also transported into plasma [25], and as a consequence, plasma acylcarnitine levels can serve as an indicator of mitochondrial function [36].

Despite the known relationship between acylcarnitine homeostasis and mitochondrial function, investigations of acylcarnitines have focused primarily on diseased populations, and little attention has been given to differences in acylcarnitine homeostasis between healthy men and women. The current study was designed to test whether acylcarnitines vary by age and gender in a healthy, non-diseased population. Results from high-resolution metabolomics (HRM) analyses show that LC and very long-chain (VLC) acylcarnitines increase with age and have gender-specific differences in healthy individuals.

## RESULTS

### Study population demographics

**Figure 1:**
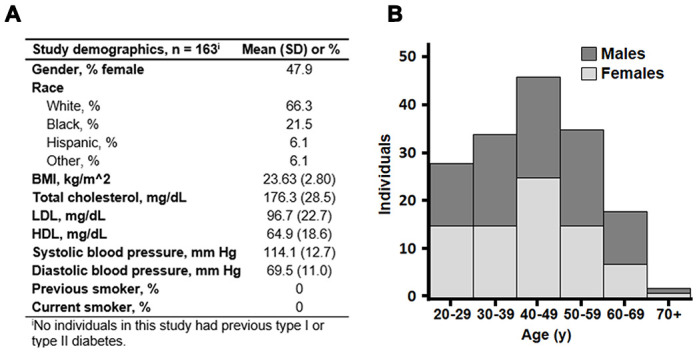
**Background characteristics of 163 healthy adults of the predictive medicine cohort.** (**A**) Mean values with standard deviation (SD) of gender, race and clinical measures are shown. (**B**) Age distribution of the subset. Stacked bars are shown with men in dark gray and women in light gray. Mean age was 43.5 years, and ages ranged from 20 to 90 years.

All 78 female adults and 85 male adults were healthy, without history of smoking, known disease or metabolic risk factors for disease (Figure 1A). The mean age was 43.5 years, ranging from 20 to 90 y (Figure 1B). The population included multiple races and ethnicities, but the population size was too small for separate analyses. There was no difference in age distribution of males and females.

### Metabolome-wide association study (MWAS) with age

**Figure 2:**
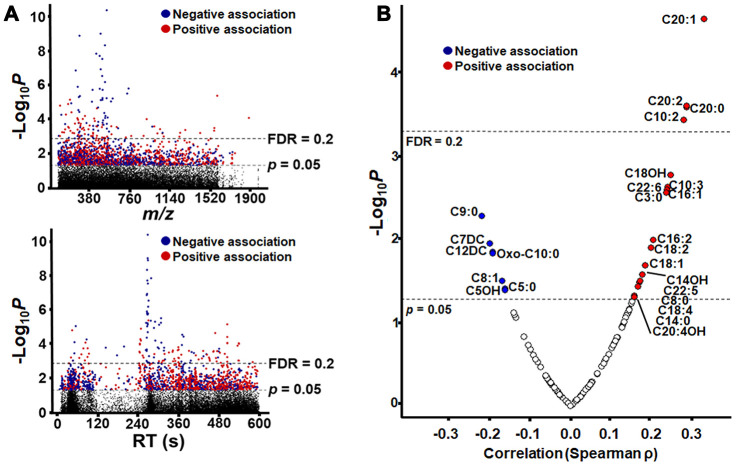
**Metabolome-Wide Association Study (MWAS) of plasma metabolites correlated with age.** (**A**) Type 1 Manhattan plot showing -log_10_ *p* for correlation of each metabolite plotted by *m/z* (mass-to-charge ratio) and type 2 Manhattan plot showing -log_10_ *p* for correlation of each metabolite plotted by chromatographic retention time (RT) in seconds, as separated the C18 column. Plots are shown with significance (n = 1505, *p* = 0.05) and false discovery rate (n= 140, FDR = 0.2) thresholds by dashed lines, and the detailed information of metabolic features is provided in Supplementary Table 1. (**B**) Plot of acylcarnitine correlation strength and direction (Spearman ρ) by –Log10 *p*. Acylcarnitines with *p* < 0.05 are labeled by the chain length, saturation and modification of the acyl group (see Table 1 for details). For acylcarnitines detected on both C18 and anion exchange columns, only the C18 data is represented in the plot. The plot is shown with significance (n = 26, *p* = 0.05) and false discovery rate (n= 4, FDR = 0.2) thresholds by dashed lines In all plots, significant negative correlations are shown in blue, and significant positive correlations are shown in red.

After filtering for *m/z* features present in at least 80% of samples, 26045 features (hereafter referred as “metabolic features” or “metabolites”) were detected by C18 and anion exchange (AE) columns, with 25680 unique *m/z* within 10 ppm. MWAS (*p* < 0.05) showed that 1915 features associated with age (154 features at FDR <0.2), with 986 associating positively and 929 associating negatively (Figure 2A). In the current study, we retained all features with p < 0.05 for annotation of possible acylcarnitines and subsequent analysis. Full listings of these are provided (C18, Supplementary Table 1; AE, Supplementary Table 2) and respective annotations are given (Supplementary Tables 3 and 4).

**Table 1:** Correlation of acylcarnitines with age.

***m*/*z***	**RT (s)**	**SLC**	**Database**	**Common name**	**Median intensity**	**Correlation (ρ)**	***p*-value**
C18 positive							
162.1116	42	2b	HMDB00062	Carnitine (C0)	3.60E6	-0.0437	0.5797
454.3869	470	3	LMFA07070010	Eicosenoylcarnitine (C20:1)	2.60E5	0.3249	2.32E-5
452.3712	391	3	LMFA07070011	Eicosadienoylcarnitine (C20:2)	1.78E5	0.2827	2.56E-4
456.4025	465	3	HMDB06460	Arachidyl carnitine (C20:0)	9.27E4	0.282	2.65E-4
312.2154	93	3	HMDB13325	Decadienoylcarnitine (C10:2)	3.01E5	0.2754	3.73E-4
444.3665	348	3	LMFA07070028	Hydroxystearoylcarnitine (C18OH)	7.27E4	0.2439	1.70E-3
310.1998	76	3	LMFA07070016	Decatrienoylcarnitine (C10:3)*	1.03E6	0.2363	2.39E-3
472.3387	335	3	HMDB06510	Docosahexaenoylcarnitine (C22:6)	2.42E4	0.2344	2.60E-3
398.3245	334	3	HMDB06317	Hexadecenoylcarnitine (C16:1)*	6.73E5	0.2341	2.63E-3
218.1375	258	2b	HMDB00824	Propionylcarnitine (C3:0)	5.14E4	0.2327	2.79E-3
396.3088	313	3	HMDB13334	Hexadecadienoylcarnitine (C16:2)*	2.30E5	0.2005	0.0103
422.3244	323	3	HMDB06318	Octadecatrienoylcarnitine (C18:3)	2.19E5	0.1947	0.0128
388.3037	286	3	HMDB13166	Hydroxymyristoylcarnitine (C14OH)	1.21E5	0.1735	0.0268
474.3564	347	3	HMDB06321	Docosapentaenoylcarnitine (C22:5)	4.26E4	0.1683	0.0318
420.3089	314	3	HMDB06463	Octadecatetraenoylcarnitine (C18:4)	2.62E4	0.1553	0.0478
464.3352	309	3	LMFA07070036	Hydroxyicosatetraenoylcarnitine (C20:4OH)	4.94E3	0.1539	0.0498
262.1637	258	2b	LMFA07070041	Hydroxyvalerylcarnitine (C5OH)	1.51E4	-0.1602	0.0411
246.1687	265	2b	HMDB13128	Valerylcarnitine (C5:0)*	5.07E4	-0.1612	0.0398
374.2519	311	3	HMDB13327	Dodecanedioylcarnitine (C12DC)	8.53E4	-0.1914	0.0144
304.1741	257	3	HMDB13328	Pimelylcarnitine (C7DC)	2.97E4	-0.1976	0.0114
302.231	301	3	HMDB13288	Nonanoylcarnitine (C9:0)	6.07E4	-0.2174	5.30E-3
Anion exchange							
310.2014	57	3	LMFA07070016	Decatrienoylcarnitine (C10:3)*	3.30E5	0.2421	1.85E-3
398.3265	76	3	HMDB13207	Hexadecenoylcarnitine (C16:1)*	3.68E5	0.1945	0.0128
426.3576	111	3	HMDB06351	Octadecenoylcarnitine (C18:1)	2.55E6	0.1808	0.0209
396.3108	65	3	HMDB13334	Hexadecadienoylcarnitine (C16:2)*	1.21E5	0.1723	0.0279
369.2828	59	3	HMDB13331	Tetradecadienoylcarnitine (C14:2)	1.20E5	0.1682	0.0318
288.2169	63	2b	HMDB00791	Octanoylcarnitine (C8:0)	5.17E5	0.1632	0.0374
372.3109	71	3	HMDB05066	Myristoylcarnitine (C14:0)	2.51E5	0.1547	0.0486
286.2013	570	2b	HMDB13324	Octenoylcarnitine (C8:1)	2.72E4	-0.1681	0.032
246.1698	581	2b	HMDB13128	Valerylcarnitine (C5:0)*	7.13E3	-0.1778	0.0232
330.2272	584	3	HMDB13202	Ketodecanoylcarnitine (Oxo-C10)	2.08E3	-0.1905	0.0149

Correlation analyzed by Spearman (ρ) and significance of correlation (*p*-value) are shown for each acylcarnitine detected on both C18 and anion exchange columns. Mass-to-charge ratio (*m/z*), retention time (RT), Schymanski level of confidence (SLC), and Human Metabolome Database (HMDB) or Lipid Maps (LMFA) database identifier are given for each acylcarnitine. Although no significant correlation was found for free carnitine (C0), values are given for reference. A SLC of 3 indicates a putative identification using LCMS data, whereas a SLC of 2b indicates a probable match using additional diagnostic MS/MS data. All acylcarnitines were detected as an M + H adduct. *, acylcarnitine identified by both C18 and anion exchange.

Annotation of features with accurate mass match to M + H adducts of acylcarnitines with *xMSannotator* [37] resulted in 132 annotated acylcarnitines, 30 of which displayed a significant association with age (*P*<0.05, Table 1). For some of the higher abundance features, MS/MS spectra showed nominal mass fragments at 85 and 144, characteristic of acylcarnitines, and the features are therefore discussed as the corresponding acylcarnitines with accurate mass match (see also Figure 5, below). Among the *m/z* features matching acylcarnitines, 4 were identified by both C18 and AE columns, resulting in 26 uniquely annotated acylcarnitines that varied with age. Nineteen of these correlated positively with age, and 7 correlated negatively with age (Figure 2B). Carnitine (*m/z* 162.1116, 42 s) did not vary with age (*p* = 0.58).

### Acylcarnitine associations with age

**Figure 3:**
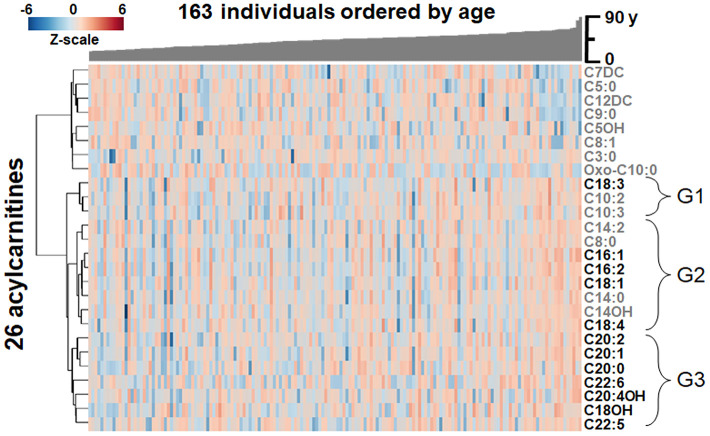
**Heat map of one-way hierarchical clustering analysis (HCA) of the 26 acylcarnitines significantly associating with age.** Along the x-axis, individuals are organized by age, with youngest on the left. The y-axis is comprised of the one-way HCA of acylcarnitines. Each column represents an individuals’ metabolic profile of the 35 acylcarnitines. Degree of deviation of acylcarnitine concentration below the mean of the study population are indicated by saturation of blue coloration, and degree of deviation of acylcarnitine concentration above the mean of the study population are indicated by saturation of red coloration. Short-chain and medium-chain acylcarnitines are labeled in gray, and long-chain and very-long-chain acylcarnitines are highlighted by labeling in black. For acylcarnitines detected on both C18 and anion exchange columns, only the C18 data was included in the HCA. The lower major acylcarnitine cluster is labeled by its subgroups, G1-3.

The 26 acylcarnitines found to associate with age in plasma were compared for similarity between individuals through one-way hierarchical clustering analysis (HCA), with the study population arranged by age (Figure 3). Results showed LC and VLC acylcarnitines (≥ 16 carbon chain) [38] (Figure 3) clustered together in a major lower cluster and several medium-chain (MC) and short-chain (SC) acylcarnitines clustered into an upper cluster (see also Figure 2B).

The lower cluster of 12 LC and VLC acylcarnitines contained three subclusters, labeled G1-G3 in Figure 3. G1 contained C18:3 as well as C10:2 and C10:3 (Figure 3). G2, the largest of the three, contained 8 acylcarnitines with 14- and 16-carbon acyl groups as well as C8:0, C18:4 and C18:1 (Figure 3). G3 contained 7 acylcarnitines with 20- and 22-carbon acyl groups as well as C18OH (Figure 3). In all three subgroups, abundances of LC and VLC acylcarnitines were increased with age.

**Figure 4:**
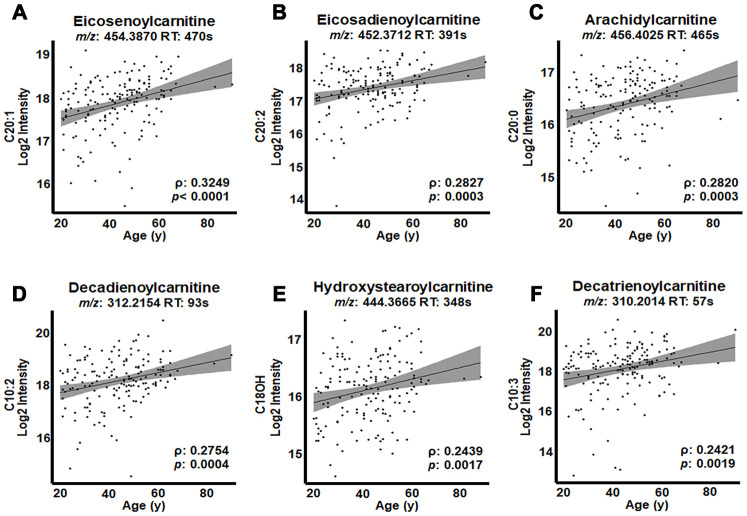
**Highest correlations of acylcarnitines with age in human plasma.** Log2 transformed intensity values for ions, identified by mass-to-charge ratio (*m/z*) and retention time (RT) for individual plasma samples are plotted against individual ages. Confidence intervals (95%) are shown in gray. (**A**) Eicosenoylcarnitine (C20:1), (**B**) eicosadienoylcarnitine (C20:2), (**C**) arachidylcarnitine (C20:0) and (**D**) decadienoylcarnitine (C10:2) were significant at FDR = 0.2. (**E**) Hydroxystearoylcarnitine (C18OH) and (**F**) decatrienoylcarnitine (C10:3) were significant at *p* < 0.05.

The top three acylcarnitines by strength of association were present in G3 and each had 20-carbon acyl groups: eicosenoylcarnitine (C20:1; ρ = 0.3249, *p* < 0.0001; Figure 4A), eicosadienoylcarnitine (C20:2; ρ = 0.2827, *p* = 0.0003; Figure 4B) and arachidylcarnitine (C20:0; ρ = 0.2820, *p* = 0.0003; Figure 4C). The following three acylcarnitines by strength of association were present in G2 and included decadienoylcarnitine (C10:2; ρ = 0.2754, *p* = 0.0004; Figure 4D), hydroystearoylcarnitine (C18OH; ρ = 0.2439, *p* = 0.0017; Figure 4E) and decatrienoylcarnitine (C10:3; ρ = 0.2421, *p* = 0.0019; Figure 4F). The relative intensity values (Table 1) showed that the most highly associated acylcarnitines had relatively high abundances compared to other acylcarnitines associated with age.

**Figure 5:**
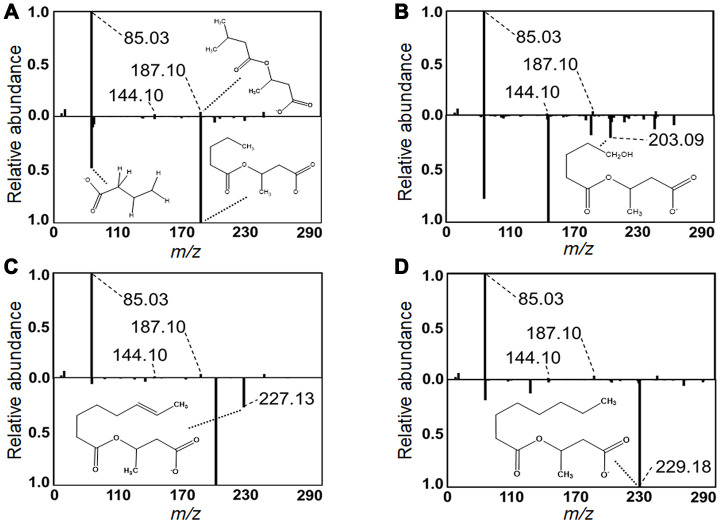
**Identification of acylcarnitines by MS/MS.** Experimental MS/MS fragmentations of (**A**) valerylcarnitine, (**B**) hydroxyvalerylcarnitine, (**C**) octenoylcarnitine and (**D**) octanoylcarnitine are juxtaposed below a library MS/MS fragmentation of methylbutyroylcarnitine. Diagnostic fragments common between library and experimental fragmentations are labeled. Pertinent MS/MS peaks are labeled for mass-to-charge ratio (*m/z*) by broken line. Additionally, distinctive fragments equivalent in mass difference to that of the mass difference between the represented acylcarnitine and methylbutyroylcarnitine are labeled with proposed fragment structure displayed. MS/MS peaks and matching proposed fragment structure are labeled by dotted line.

In contrast to the pattern of the LC and VLC acylcarnitines, which increased with age, the MC and SC cluster decreased in association with age (Figures 2B, 3). These were all relatively low abundance signals (Table 1). The MC and SC cluster included odd-chain (OC; C5, C7, C9) acylcarnitines, all of which decreased in association with age (Figure 3). The MC and SC cluster also contained features matching acylcarnitine derivatives of two dicarboxylic acids (DC) (Figures 2B, 3). Propionylcarnitine (C3:0) was present in the MC and SC cluster but did not decrease with age unlike the other OC acylcarnitines (see also Figure 2B). MS/MS fragmentation supported the identity of valerylcarnitine (C5:0; Figure 5A), hydroxyvalerylcarnitine (C5OH; Figure 5B), octenoylcarnitine (C8:1; Figure 5C) and octanoylcarnitine (C8:0; Figure 5D) relative to database spectra for methylbutyroylcarnitine. C5:0 matched several fragments for methylbutyroylcarnitine (Figure 5A). C5OH, C8:1 and C8:0 matched with methylbutyroylcarnitine, with the exception of one major fragment. The mass difference for fragments matched the mass differences present between the given acylcarnitine and methylbutyroylcarnitine (Figure 5B–5D).

Tests for correlations among the acylcarnitines in Figure 3 showed that the high-abundance LC and VLC metabolites in G3 were highly positively correlated with each other, with the one exception of C22:6, which did not correlate with any other LC or VLC acylcarnitines (Supplementary Table 5). In contrast, these LC and VLC acylcarnitines were not correlated, either positively or negatively, with carnitine or acetylcarnitine. Propionylcarnitine was associated with arachidylcarnitine C20:0 (ρ = 0.2968, *p* = 0.0001). Few associations occurred among MC and SC acylcarnitines (Supplementary Table 5). Acylcarnitines with a 10-carbon acyl group as well as C14OH had associations with LC and VLC acylcarnitines (Supplementary Table 5). OC acylcarnitines had few correlations; only C9:0 correlated with C5:0 (ρ = 0.3561, *p* < 0.0001) and C7DC (ρ = 0.3583, *p* < 0.0001; Supplementary Table 5). Hydroxyvalerylcarnitine (C5OH) showed no correlation with any other OC acylcarnitines.

### Gender differences in acylcarnitine associations with age

The 6 acylcarnitines with greatest positive rho value were examined for their associations with age when individuals were separated by gender. This separation by gender did not show stronger correlation of acylcarnitines with age (Table 2) than observed for the combined analyses (Fig 5). Comparisons of genders show that C20:1 and C20:2 exhibited stronger correlations with age in males than females while C20:0, C10:2, C18OH and C10:3 exhibited slightly stronger correlations with age in females than males.

**Table 2:** Comparison of correlations of acylcarnitines with age by gender.

**Name**	**Structure**	**Female (*ρ*, *p*)**	**Male (*ρ*, *p*)**
Eicosenoylcarnitine	C20:1	0.2828, **0.0121**	0.3551, **8.55E-4**
Eicosadienoylcarnitine	C20:2	0.1344, 0.2409	0.3693, **5.04E-4**
Arachidylcarnitine	C20:0	0.3078, **6.11E-3**	0.2397, **0.0271**
Decadienoylcarnitine	C10:2	0.3194, **4.37E-3**	0.2323, **0.0324**
Hydroxystearoylcarnitine	C18OH	0.3455, **2.95E-3**	0.1457, 0.1845
Decatrienoylcarnitine	C10:3	0.2456, **0.0330**	0.2304, **0.0365**

For the 6 acylcarnitines with the highest correlation with age in the whole population, correlation with age by gender was tested. Spearman’s ρ and *p*-value are shown for correlations in females and males.

**Figure 6:**
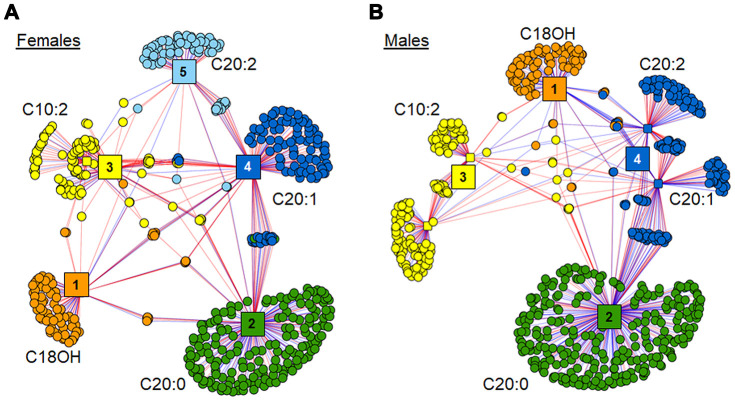
**Association of top 6 age-associated acylcarnitines with metabolome.** (**A**) *xMWAS* network of top 6 age-associated acylcarnitines as associated with metabolome within females. Cluster 1 (orange) has features predominantly associated with hydroxystearoylcarnitine (C18OH). Cluster 2 (green) is comprised of features associated most closely with arachidylcarnitine (C20:0). Cluster 3 (yellow) contains features clustered around decadienoylcarnitine (C10:2) and decatrienoylcarnitine (C10:3). Cluster 4 (dark blue) has features mainly associated with eicosenoylcarnitine (C20:1). Cluster 5 (light blue) has features mainly associated with eicosadienoylcarnitine (C20:2). See Supplementary Table 6 for detailed annotation of metabolites included in the female network. (**B**) *xMWAS* network of top 6 age-associated acylcarnitines as associated with metabolome within males. Clusters 1-3 form around the same acylcarnitines as their respective acylcarnitines in the female subset. Cluster 4 (blue) has features mainly associated with both C20:1 and C20:2. See Supplementary Table 7 for detailed annotation of metabolites included in the male network. Positive associations are shown in red, while negative associations are shown with blue lines.

The *xMWAS* analysis of these 6 acylcarnitines against the remainder of the metabolome in each gender resulted in different clustering within each network (Fig 6). The female network grouped into 5 distinct clusters, with separate clusters for C18OH, C20:0, C20:1 and C20:2 (Clusters 1, 2, 4 and 5, respectively), and C10:2 and C10:3 (Cluster 3) were consolidated into one cluster of metabolites (Figure 6A). Relative to females, the male network grouped more tightly resulting in 4 clusters, with C20:1 and C20:2 in Cluster 4 (Figure 6B). Additionally, the three 20 carbon acylcarnitines showed associations with more metabolites in the males compared to females.

Metabolites associated with these 6 acylcarnitines shared little commonality between females and males, with less than 11% of those metabolites included in the female network also being present in the male network. Annotations of the features in the female and male *xMWAS* networks are provided in Supplementary Tables 6 and 7, respectively. Pathway enrichment analyses of the metabolic features associated in both the female and male *xMWAS* networks resulted in distinctly different pathways associating with age-associated acylcarnitines in women and in men. Cytochrome P450 metabolism (females: *p* = 0.001; males: *p* = 0.030) and vitamin E metabolism (females: *p* = 0.006; males: *p* = 0.001) changed in association with age-associated acylcarnitines in both genders (Table 3). In females, glycerophospholipid metabolism (*p* = 0.004), leukotriene metabolism (*p* = 0.004), fatty acid activation (*p* = 0.017), glycosphingolipid metabolism (*p* = 0.020), prostaglandin formation (*p* = 0.028), tryptophan metabolism (*p* = 0.032) and fatty acid biosynthesis (*p* = 0.039) changed in association with age-associated acylcarnitines (Table 3). In males, the carnitine shuttle (*p* = 0.002), cholesterol biosynthesis (*p* = 0.011) and steroid biosynthesis (*p* = 0.030) changed in association with age-associated acylcarnitines (Table 3).

**Table 3:** Metabolic pathways associated with top 6 age-related acylcarnitines by gender.

**Metabolic Pathway**	**Number of metabolites at *p* < 0.05**	**Number of metabolites from pathway detected**	***p*-value**
*Females*			
Cytochrome P450 drug metabolism	5	26	0.001
Glycerophospholipid metabolism	4	28	0.004
Leukotriene metabolism	6	51	0.004
Vit E metabolism	4	30	0.006
Fatty acid activation	3	24	0.017
Glycosphingolipid metabolism	3	25	0.020
Prostaglandin formation	4	40	0.028
Tryptophan metabolism	4	41	0.032
Fatty acid biosynthesis	3	29	0.039
*Males*			
Vit E metabolism	6	30	0.001
Carnitine shuttle	5	28	0.002
Cholesterol biosynthesis	4	31	0.011
Steroid biosynthesis	5	53	0.030
Cytochrome P450 drug metabolism	3	25	0.030

## DISCUSSION

Our results show that plasma LC and VLC acylcarnitines increase with age in healthy individuals. In individuals with no age-related disease nor metabolic risk factors, all age-associated acylcarnitines with acyl groups of carbon chains 16 carbons or longer increased in association with age. All OC acylcarnitines except for C3:0 were decreased with age. The results also show that among the most strongly age-associated acylcarnitines, C20:2 and C18OH show difference in strength of association between genders, and the genders differ considerably in lipid pathways which vary in association with age-associated acylcarnitines.

Changes in acylcarnitines with age alone point toward decreased mitochondrial function with age. Elevated acylcarnitines, especially LC and VLC acylcarnitines, serve as markers of mitochondrial deficiency in fatty acid oxidation [25, 26]. Downstream associations of increased LC acylcarnitines with other lipid metabolic pathways, such as glycerophospholipid, glycosphingolipid, fatty acid and cholesterol metabolism, further suggests that the effect of age occurs through changes in mitochondrial function [39]. Inverse association of hydroxyvalerylcarnitine (C5OH) with age as well as the lack of association of C5OH with any other age-related OC acylcarnitines suggests changes in other mitochondrial pathways such as branched chain amino acid (BCAA) metabolism [40, 41]. Negative association of dicarboxylic acylcarnitines (C7DC and C12DC) with age suggests decreased β-oxidation of LC dicarboxylic acylcarnitines in the peroxisome with age [42]. Increased LC and VLC with age and dysregulation of BCAA metabolism and mitochondrial and peroxisomal lipid metabolism are consistent with the effect of age being upstream of the mitochondria. Such upstream effects on mitochondrial lipid and amino acid metabolism and peroxisomal activity are known to occur through changes in mTOR/PPARα activity with age [43, 44].

The increase in acylcarnitines in healthy individuals with increased age alone suggests a need for improved matching of case and control groups in studies of age-related disease. For instance, in some studies on increased acylcarnitines in age-related disease, case groups were older than controls [12–14, 23]. Other studies used populations in which there was no significant difference in age of cases and controls, but the case group mean age was over 5 years greater than that of the control group [16, 19, 22]. The consistency of these studies with regard to associations of acylcarnitines and disease supports the correctness of the authors conclusions; none-the-less, the magnitude of effects might be impacted by non-disease-related, age-associated changes of acylcarnitines in the controls.

Two LC acylcarnitines, C16:1 (hexadecenoylcarnitine) and C18:1 (octadecenoylcarnitine), positively correlated with age in the present study, have been reported to be positively associated with age-related disease in some of these studies. Bouchouirab et al. [13] reported decreased plasma clearance of C18:1 in response to insulin or postprandially in individuals with type II diabetes when compared to controls who were non-diabetic with no family history of diabetes. In this study, the mean age of individuals with type II diabetes was nearly twice that of the controls group. Similarly, Adams et al. [16] reported increased fasting C18:1 in obese, African American women with type II diabetes compared to that of obese, African American women without disease. In this study, the mean age of diabetic individuals was 5 years older than that of controls, and the maximum age studied for the diabetic group was 18 years greater than that of the control group. Lastly, a recent study of neovascular age-related macular degeneration [14] reported increased C16:1 and C18:1 in diseased individuals compared to controls. Their study controlled for age in the analysis, and the mean age for both groups was above 70 years old. Our present study mostly had individuals 20-70 years of age, and this limits conclusions which may be drawn from comparisons.

The difference in direction of association of C3:0 compared to all other OC acylcarnitines may be a result of decreased flux with age because propionyl-CoA is a product of OC fatty acid β-oxidation [45]. Increased C3:0 with age may be indicative of an age-related change in metabolism of OC LC fatty acids, which have been shown to associate negatively with heart disease and type II diabetes [46, 47]; however, we observed no association of OC LC acylcarnitines with age. Alternatively, the decrease in C5:0, C7DC and C9:0 with age may be a result of decreased dairy consumption with age, as dairy products are an important source of OC fatty acids in the diet [48, 49]. This increase in C3:0 with age could also be due to age-related differences in composition of the microbiome or in dietary fiber intake [50]; these parameters were not assessed in our study population. Future study of metabolic flux using stable isotope-labeled OC fatty acids could shed light on whether this observation is due to an age-related change in metabolism or an external factor such as dietary fiber or dairy intake.

As with previously noted differences in acylcarnitine association with age in men and women, we also found differences in strength of association of several acylcarnitines with age between genders. Previously, Muilwijk et al. [33] reported an increase in concentrations of several acylcarnitines with age in individuals who were without age-related disease but whose clinical measures were not controlled. They noted that increases observed in acylcarnitines with age were higher in women, and several more significant changes in acylcarnitines with age were reported for women than were for men. Yu et al. [32] reported C18:1 as a correlate with age in both men and women; however, in women the relationship was much stronger. In neither of these studies were the differences in strengths of correlation a focus. Similarly, our data show that there is difference between the genders in how strongly acylcarnitines associate with age. Our study shows different associations of acylcarnitines with the remainder of metabolism. Generally, women exhibited more lipid metabolic pathways which were altered in association with age-associated acylcarnitines. In addition to this, women exhibited changes in leukotriene metabolism, prostaglandin formation and tryptophan metabolism in association with age-associated acylcarnitines. Association of these inflammatory-related pathways with age-associated acylcarnitines in women but not in men support previous findings that gender differences in inflammatory pathways tend to magnify in old age [51–54].

The present study used stringent selection criteria for health of individuals studied; however, the population was small and cannot be considered representative of the general population, especially for racial and ethnic comparisons. Additionally, this was a cross-sectional study of individuals at different ages and does not address longitudinal changes within individuals as a result of aging. Studies are needed to evaluate changes in acylcarnitines which occur as a result of loss of mitochondrial function and other physiological measures of aging, such as telomeric shortening, loss of proteostasis, deregulated nutrient-sensing or physical frailty [55, 56].

In summary, the present results show that abundant acylcarnitines increase in plasma with age in healthy individuals. The results emphasize the importance of strict control for age in metabolomic studies of age-related diseases in order to account for metabolic alterations which occur as an adaptive response in healthy aging regardless of disease state. The study also shows important gender differences in glycerolipids and other metabolic networks linked to acylcarnitines that vary by age. Thus, the results provide justification for detailed studies of lipid metabolism in aging, specifically to understand gender differences which could impact underlying gender-specific disease mechanisms.

## MATERIALS AND METHODS

### Chemicals

HPLC grade acetonitrile and methanol, LC-MS water and 98% formic acid were obtained from Sigma-Aldrich (St. Louis, MO). A mixture of 14 stable isotopic chemicals used as an internal standard [57] included [^13^C_6_]-D-glucose, [^15^N]-indole, [2-^15^N]-L-lysine dihydrochloride, [^13^C_5_]-L-glutamic acid, [^13^C_7_]-benzoic acid, [3,4-^13^C_2_]-cholesterol, [^15^N]-L-tyrosine, [trimethyl-^13^C_3_]-caffeine, [^15^N_2_]-uracil, [3,3-^13^C_2_]-cystine, [1,2-^13^C_2_]-palmitic acid, [^15^N,^13^C_5_]-L-methionine, [^15^N]-choline chloride and 2’-deoxyguanosine-^15^N_2,_^13^C_10_-5’-monophosphate from Cambridge Isotope Laboratories, Inc (Andover, PA).

### Human plasma samples

A subset of samples (n = 163) from the Predictive Medicine Research (PREMED) cohort (ClinicalTrials.gov Identifier: NCT00336570) was used; the subset represented all available samples and did not appear to have selection bias. The study was reviewed and approved by the Emory University Investigational Review Board (IRB00024767). PREMED subjects were healthy individuals between 20 and 90 years of age. Participants were originally studied to define a “normal” clinical value or range of values for plasma contents in healthy individuals to evaluate methods for detecting early multiorgan disease (NCT00336570). Included participants possessed low BMI, LDL cholesterol and blood pressure as well as no history of smoking. Participants were excluded by use of lipid lowering medication, presence of any number of chronic or acute diseases or disorders, listed in full detail at ClinicalTrials.gov. Blood plasma was collected with EDTA, and samples were stored at -80°C prior to LC-MS analysis.

### High-resolution metabolomics (HRM)

Plasma samples were analyzed as described previously [58]. Briefly, 50 μL plasma samples were treated 2:1 (v/v) with acetonitrile, and 2.5 μL internal standard of a mixture of 14 stable isotope standards was added. Proteins were precipitated and pelleted by incubation at 4°C for 30 min followed by centrifugation for 10 min at 21000 x *g* at 4°C. Supernatants were placed in autosampler vials and maintained at 4°C in an autosampler. Samples were analyzed by liquid chromatography-Fourier transform mass spectrometry at 60,000 resolution (Accela-LTQ Velos Orbitrap; *m/z* range from 85-2000) in triplicate. A dual chromatography setup was utilized, using AE and C18. Electrospray ionization was performed in positive ion mode. Data collection occurred continuously throughout 10 min of chromatographic separation.

Raw files were converted to .cdf files using Xcalibur file converter from Thermo Fisher (San Diego, CA). Data extraction was performed using *apLCMS* [59] and *xMSanalyzer* [60], generating *m/z* features, a *m/z* feature consisting of mass-to-charge ratio (*m/z*), retention time (RT) and ion intensity. Feature and sample filtering retained features with a median CV of 50% or less, a minimum mean Pearson correlation coefficient of 0.7 between technical replicates of each sample, and which were detected in at least 30% of samples. Two pooled human reference plasma samples were concurrently analyzed, as described by [61]. Briefly, NIST SRM1950 was analyzed at the beginning and end of the study. A second pooled reference sample (Q-Standard 3; Qstd3) was analyzed at the beginning of each batch of 20 samples. Qstd3 was prepared from plasma pooled from 2 separate lots from Equitech-Bio, Inc (Kerrville, Texas).

### Metabolite annotation and identification

The *m/z* features were annotated for possible identities against the Human Metabolome Database (http://www.hmdb.ca/) and the LIPID MAPS Lipidomics Gateway (http://www.lipidmaps.org/) using *xMSannotator* [37], which scores annotations based on correlation modularity clustering and isotopic, adduct and mass defect grouping. This provides annotation at a confidence equivalent to a Schymanski level of confidence (SLC) 3 as defined by Schymanski et al. [62]. All metabolites were matched at < 10 ppm accuracy. All acylcarnitines were detected in the form of a M + H adduct. All age-associated acylcarnitines were tested for probable structure using MS/MS fragmentation with the Accela-LTQ Velos Orbitrap. Acylcarnitines for which probable structure could be identified by diagnostic fragments compared against values from the METLIN MS/MS metabolite database [63] were designated as SLC 2b as defined by Schymanski et al. [62].

### Metabolome-wide association study of age

Using the R package, *xmsPANDA* (https://github.com/kuppal2/xmsPANDA), a metabolome-wide association study (MWAS) was performed to identify *m/z* features which associate with age in healthy individuals. Intensities for *m/z* features from *xMSanalyzer*, which had intensities for at least 80% of samples were log2 transformed and quantile normalized. Missing values were replaced by half of the minimum reported intensity for that feature. Normalized *m/z* features were tested by Spearman’s rank correlation for association with age, with a *p* < 0.05 considered significant. A Benjamini/Hochberg false discovery rate (FDR) method was used to correct for multiple comparisons, with an FDR threshold of 0.2 [64]. Hierarchical clustering analysis (HCA) was performed using MetaboAnalyst [65].

All acylcarnitines found to associate with age from the C18 column were analyzed for correlation between acylcarnitine subtypes. Age-associated acylcarnitines from the C18 column were chosen over those from the AE column due to greater coverage of acylcarnitines. In addition to those found to be age-associated, free carnitine (C0) and acetylcarnitine (C2) were included for analysis. Correlations were analyzed using Spearman’s rank correlation test, and a Bonferroni correction was applied [66], resulting in *p* < 1.98 * 10^4^ being considered significant.

The top 6 age-associated acylcarnitines were tested for associations with other components of the metabolome in each gender using *xMWAS* based on partial least-squares regression [67]. After using the data normalization and filtering methods described above, the top 6 age-associated acylcarnitines were tested for associations with 26040 metabolites within both female (n = 78) and male (n = 85) subsets of the population. In generation of both networks, metabolites were filtered so that the 10000 metabolites with the lowest relative standard deviation were included in the partial least squares analysis. Thresholds for inclusion in the network were |r| > 0.30 and *p* < 0.01. Pairwise results from MWAS of the metabolites used for generation of network structures were used for pathway enrichment analysis using *mummichog (v1)* [68]. For each gender, enriched pathways were filtered for those that included at least 3 significantly associated metabolites at *p* < 0.05.

## Supplementary Material

Supplementary Table 1

Supplementary Table 2

Supplementary Table 3

Supplementary Table 4

Supplementary Table 5

Supplementary Table 6

Supplementary Table 7
