# Supplementary figures and images for: Plasma acylcarnitine levels increase with healthy aging
Source: Aging (Albany NY). 2020 Jun 16;12(13):13555–70. doi: 10.18632/aging.103462 (PMC7377890; doi:10.18632/aging.103462)

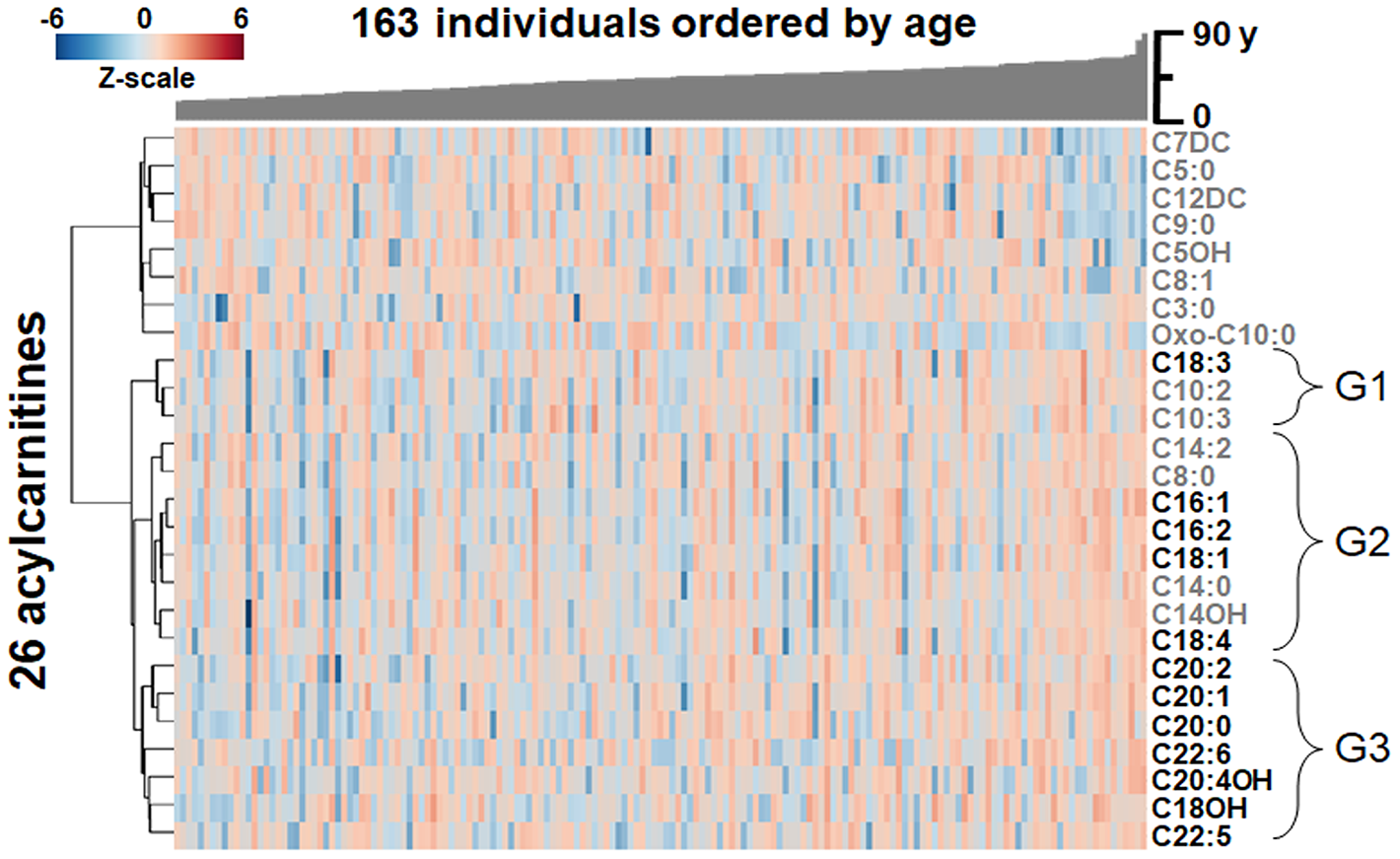

Supplement: Supplementary Table 3 [file aging-12-103462-s007..tif]

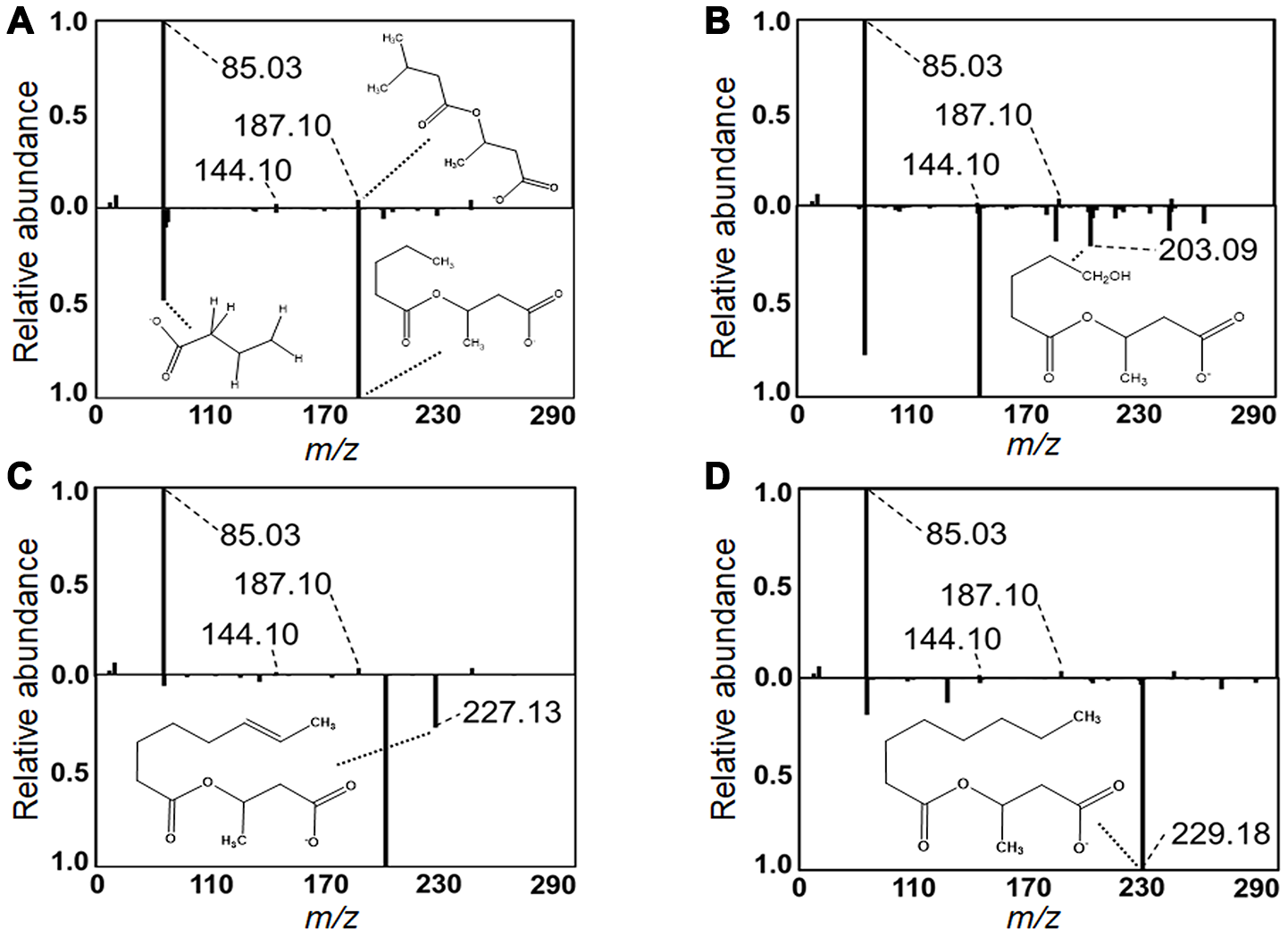

Supplement: Supplementary Table 5 [file aging-12-103462-s006..tif]
